# Comprehensive Landscape of Active Deubiquitinating Enzymes Profiled by Advanced Chemoproteomics

**DOI:** 10.3389/fchem.2019.00592

**Published:** 2019-08-29

**Authors:** Adán Pinto-Fernández, Simon Davis, Abigail B. Schofield, Hannah C. Scott, Ping Zhang, Eidarus Salah, Sebastian Mathea, Philip D. Charles, Andreas Damianou, Gareth Bond, Roman Fischer, Benedikt M. Kessler

**Affiliations:** ^1^University of Oxford, Oxford, United Kingdom; ^2^Target Discovery Institute, Nuffield Department of Medicine, University of Oxford, Oxford, United Kingdom; ^3^Christ Church, University of Oxford, Oxford, United Kingdom; ^4^Ludwig Institute for Cancer Research, University of Oxford, Oxford, United Kingdom; ^5^Department of Chemistry, University of Oxford, Oxford, United Kingdom; ^6^Structural Genomics Consortium (United Kingdom), Oxford, United Kingdom; ^7^Institute of Pharmaceutical Chemistry, Buchmann Institute for Molecular Life Sciences, Goethe University Frankfurt, Frankfurt, Germany

**Keywords:** deubiquitylating enzymes, mass spectrometry, proteomics, chemical biology, ubiquitin specific proteases, isoforms

## Abstract

Enzymes that bind and process ubiquitin, a small 76-amino-acid protein, have been recognized as pharmacological targets in oncology, immunological disorders, and neurodegeneration. Mass spectrometry technology has now reached the capacity to cover the proteome with enough depth to interrogate entire biochemical pathways including those that contain DUBs and E3 ligase substrates. We have recently characterized the breast cancer cell (MCF7) deep proteome by detecting and quantifying ~10,000 proteins, and within this data set, we can detect endogenous expression of 65 deubiquitylating enzymes (DUBs), whereas matching transcriptomics detected 78 DUB mRNAs. Since enzyme activity provides another meaningful layer of information in addition to the expression levels, we have combined advanced mass spectrometry technology, pre-fractionation, and more potent/selective ubiquitin active-site probes with propargylic-based electrophiles to profile 74 DUBs including distinguishable isoforms for 5 DUBs in MCF7 crude extract material. Competition experiments with cysteine alkylating agents and pan-DUB inhibitors combined with probe labeling revealed the proportion of active cellular DUBs directly engaged with probes by label-free quantitative (LFQ) mass spectrometry. This demonstrated that USP13, 39, and 40 are non-reactive to probe, indicating restricted enzymatic activity under these cellular conditions. Our extended chemoproteomics workflow increases depth of covering the active DUBome, including isoform-specific resolution, and provides the framework for more comprehensive cell-based small-molecule DUB selectivity profiling.

## Introduction

Ubiquitin (Ub) is a conserved, globular protein consisting of 76 amino acids that can be attached to proteins either in a mono- or polymerized form, impacting on their activity, localization, interactome, and turnover. The covalent attachment of Ub, most frequently to a ε-NH_2_ lysine side chain of protein substrates, is catalyzed by the sequential action of three enzymes: E1 activating enzyme, E2 conjugating enzyme, and E3 ligase (Hershko and Ciechanover, 1998). Polymers of Ub can be formed by the addition of one or more monomers to a previously substrate-attached Ub molecule. These chains provide a code of functional modulations including protein degradation and cellular signaling (Komander and Rape, 2012). Poly-Ub chains can also include Ub-like modifiers (UBLs) (Cappadocia and Lima, 2018) and posttranslational modifications (PTMs) that increase the biological complexity of ubiquitylation (Swatek and Komander, 2016).

Polymerization of Ub is a reversible process carried out by deubiquitylating enzymes (DUBs) that catalyze hydrolysis of Ub–substrate isopeptide bonds (Komander et al., 2009). To date, there are 102 human DUBs that have been grouped into eight different sub-families (Komander et al., 2009; Fraile et al., 2012): Ub-specific proteases (USPs), Ub carboxy-terminal hydrolases (UCHs), ovarian tumor domain containing proteases (OTUs), Machado–Joseph disease protein domain proteases (MJDs or Josephins), JAMM/MPN domain-associated metallopeptidases (JAMMs), motif interacting with Ub-containing novel DUB (MINDYs) (Abdul Rehman et al., 2016), the less studied monocyte chemotactic protein-induced protein (MCPIPs) (Kolattukudy and Niu, 2012), and the recently discovered Zn-finger and UFSP domain protein (ZUFSP) (Haahr et al., 2018; Hermanns et al., 2018; Hewings et al., 2018). Most DUBs (~80) are classified as cysteine proteases with the exception of JAMM metallopeptidases and inactive (pseudo) DUBs (Nijman et al., 2005; Komander et al., 2009). DUBs have emerged as key enzymes for deciding the fate of most intracellular proteins regarding their function and lifespan. Highly selective and potent DUB inhibitors are now emerging (Kategaya et al., 2017; Lamberto et al., 2017; Turnbull et al., 2017; Gavory et al., 2018; Harrigan et al., 2018; Clague et al., 2019), which, in addition to PROteolysis-TArgeting chimeras (PROTACS) (Mullard, 2019), are paving the way to explore the Ub system in drug discovery development programs by modulating the turnover of key targets in the context of cancer, dementia, and inflammation (Pinto-Fernandez and Kessler, 2016; Harrigan et al., 2018). DUB inhibitor development has been accelerated by the application of Ub activity-based probes (ABPs) (Altun et al., 2011; Turnbull et al., 2017). ABPs contain a specificity motif that targets them to the desired enzyme/class of enzymes and a chemical moiety that reacts covalently with the active site of the enzyme. This has been applicable to study proteases that have a nucleophilic active site, mainly serine hydrolases and cysteine peptidases (Sanman and Bogyo, 2014), but also metalloproteases (Nury et al., 2013; Amara et al., 2018). For DUBs, many different probe architectures have been generated and tested with different selectivity toward DUBs, with those using a molecule of Ub as specificity motif being the more popular ones (Borodovsky et al., 2001, 2002, 2005; Hemelaar et al., 2004). However, di-Ub ABPs mimicking the different poly-Ub linkages have been generated and studied (McGouran et al., 2013), with the linear di-Ub being highly selective toward OTULIN (Weber et al., 2017). Finally, thanks to the utilization of a DUB inhibitor as specificity motif, Ward et al. (2016) managed to synthesize a permeable ABP reactive with a number of DUBs. Different C-terminal chemical moieties enabling Michael additions and nucleophilic displacements have been explored, and more recently, alkynes that react *via* a radical-based mechanism (Ekkebus et al., 2013; Hewings et al., 2017). To determine the subset of DUBs that directly react with probe in addition to binding, DUB-probe reaction centric probes were generated that enrich for Cys-reactive peptides after enzymatic digestion to map covalent sites within reactive DUBs (Hewings et al., 2018). Despite these advances, it is unclear to what extent the entire range of endogenous DUBs expressed that are active in cells are captured.

To address this, we have developed an advanced “activitomics” workflow and compared it against the DUB transcriptome and proteome expressed in MCF7 breast cancer cells. We discriminate between DUBs reactive to probe and non-reactive enzyme species through competition at the enzyme's active-site cysteine combined with quantitative chemoproteomics. The range covered by the cellular DUB activitome, transcriptome (mRNA), and deep proteome (protein) is comparable, revealing an extended landscape of the cellular DUBome.

We acknowledge that it is challenging to compare proteomics data because instrumentation, methods, and software are in constant evolution and all three are quite heterogeneous from lab to lab. In this particular study, we identified 74 DUBs whereas most previous studies reported on between 20 and 40 DUBs. A recent study by Ingrid Wertz' group reported the identification of 61 DUBs (Hewings et al., 2018). Therefore, our study represents the most comprehensive coverage reported so far.

## Materials and Methods

### Contact for Reagent and Resource Sharing

Further information and requests for resources and reagents should be directed to and will be fulfilled by the corresponding authors, BK (benedikt.kessler@ndm.ox.ac.uk) and AP-F (adan.pintofernandez@ndm.ox.ac.uk).

### Cell Lines and Reagents

Commercially purchased MCF7 (ATCC Nr HTB-22) cells were cultured in DMEM medium (GIBCO) supplemented with 10% fetal calf serum (FCS; GIBCO), 100 U/ml penicillin (SIGMA), and 100 μg/ml streptomycin (SIGMA) and maintained at 37°C in a humidified atmosphere at 5% CO_2_. Other reagents used in this study are listed in Table 1.

**Table 1:** Cell lines and reagents.

**Reagent or resource**	**Source**	**Identifier**
**ANTIBODIES**		
Mouse monoclonal antibody HA (12CA5)	Roche	#11583816001
USP7 pAb	Enzo	#BML-PW0540-0100
GAPDH loading control antibody (GA1R)	Invitrogen	#MA5-15738
Monoclonal Anti-HA-Agarose antibody produced in mouse	SIGMA	#A2095-1ML
**CHEMICALS, KITS, ENZYMES, AND OTHERS**		
Trypsin (TPCK-treated)	Worthington	#LS003740
Acid-washed glass beads	SIGMA	# G4649
Pierce™ BCA Protein Assay Kit	Thermofisher	#23225
Criterion TGX Gel, 4–15%, 18-well	Bio-Rad	#5671084
Sep-Pak C18 Plus Short Cartridge, 360 mg Sorbent per Cartridge, 55–105 μm Particle Size	Waters	# WAT020515
Chitin resin	New England Biolabs	#S6651L
PD-10 columns	GE Healthcare	#17-0851-01
2-Bromoethylamine	SIGMA	#B65705-25G
Propargylamine	SIGMA	#P50900-5G
**EXPERIMENTAL MODELS: CELL LINES**		
MCF7 cells	ATCC	HTB-22
Software and Algorithms		
MaxQuant Software (version 1.5.2)	Open source	http://www.coxdocs.org/doku.php?id=maxquant:start
Perseus Software (version 1.6.2.3)	Open source	http://www.coxdocs.org/doku.php?id=perseus:start
Prism 8	GraphPad	https://www.graphpad.com/scientific-software/prism/

### Ub-Based ABP Synthesis

The construct pTYB-HAUb, comprising the sequences of the human Ub (lacking Gly 76), an intein and a chitin binding domain, plus an HA tag, was used to synthesize HAUb75-MESNa as described previously (Borodovsky et al., 2002). Briefly, Ub–intein–chitin domain fusion protein was expressed in *Escherichia coli* (18 h induction with 0.4 mM IPTG at 17°C). Cell pellets were resuspended in 50 mM HEPES, pH 7.4, 150 mM NaCl, and 0.5 mM TCEP and lysed in a high-pressure homogenizer. The cleared cell extract was loaded onto a 15 ml chitin bead (New England Biolabs) column at a flow rate of 0.5 ml/min. The column was washed with 60 ml of lysis buffer followed by 25 ml of lysis buffer containing 50 mM β-mercaptoethanesulfonic acid sodium salt (MESNa) and incubated overnight at 37°C for the induction of on-column cleavage. HAUb75-MESNa thioester was eluted with 25 ml of lysis buffer and concentrated: approximately 2.5 mg of protein was recovered from a 1-L culture. The N-terminal Met of the HA-tag was frequently processed off during expression, resulting in a mixture of two proteins that behaved identically in labeling experiments.

To synthesize the HA-UbC2Br or HA-UbPA probes, 0.2 mM of 2-bromoethylamine or 250 mM propargylamine was added to a solution of HAUb75-MESNa (1–2 mg/ml) in 500 μl of column buffer, respectively. pH was carefully adjusted to 8 with NaOH, and after 20 min shaking at 1,400 rpm, at room temperature, 100 μl of 2.0 M aqueous HCl was added and the resultant reaction mixture was promptly transferred to a PD10 gravity column for buffer exchange, according to the manufacturer's instructions.

The probe was then aliquoted and frozen at −80°C for storage (no significant deterioration is observed for several months of storage except for HA-UbC2Br, which is prone to hydrolysis). All HA-Ub-derived probes were analyzed by liquid chromatography mass spectrometry (LC-MS) using a 1290 UPLC (Agilent) coupled to a 6560 quadrupole time-of-flight (QToF) mass spectrometer (Agilent) to monitor the reaction and the product detected by [M+H]^+^ = 10,197.6221, with >90% purity.

### Preparation of Cell Extracts and Western Blotting

Protein extracts were prepared as follows: Cells were washed with ice-cold PBS and collected into a centrifuge tube in either glass beads lysis buffer (GBL: 50 mM Tris, pH 7.5, 5 mM MgCl_2_, 0.5 mM EDTA, and 250 mM Sucrose) or glass beads lysis buffer plus 0.2% NP-40 (GBLN). One volume of acid-washed glass beads (Sigma Aldrich, G4649) per 2–3 volumes of ice-cold glass bead buffer (+1 mM DTT) was added to the tube containing the cells and buffer followed by vortexing (10 times in 30 s bursts, returning the samples to the ice for 1–2 min in between) and centrifugation (14,000 *g*, 4°C, 25 min) in order to pellet the glass beads, nuclei, and membranes. The supernatant was carefully transferred to fresh Eppendorf tubes and the pellets were discarded. The protein concentration was determined *via* the Thermo BCA protein assay kit. For Western blotting, 25 μg of protein was then fractionated on Tris–glycine SDS-PAGE gradient (4–15% acrylamide) gels, transferred onto PVDF membranes, and detected with the indicated antibodies using a LI-COR detection system.

### DUB Activity-Based Profiling

At least 500 μg of cell extract (corresponding to approximately 1 × 10^7^ cells) in 300 μl of GBL buffer containing 1 mM DTT were utilized for the ABP pulldowns. When profiling a DUB inhibitor, the inhibitor should be added at this point to the desired final concentration and incubated at 37°C for 1 h. Then, ~10 μg of the HA-Ub-based ABPs were added per 500 μg of sample (Note: this will vary depending on the reactivity of the probe batch and type and may require optimization for complete labeling of the DUB of interest) and incubated at 37°C for 45 min. The reaction was quenched by the addition of SDS to 0.4% (24 μl of a 5% stock per 300 μl) and NP-40 (or IGEPAL CA-630 substitute) to 0.5% (15 μl of a 10% stock per 300 μl), and samples were diluted to 1 ml, 0.5 mg/ml, by the addition of 661 μl of NP-40 lysis buffer [pH 7.4, 50 mM Tris, 0.5% (v/v) NP-40, 150 mM NaCl, and 20 mM MgCl_2_]. Fifty microliters (25 μg) of sample was aliquoted and denatured by boiling in SDS Laemmli sample buffer for control blotting to assess IP efficiency. To bind and pull down DUB–ABP complexes, 150 μl of anti-HA-Agarose slurry (previously washed four times with NP-40 lysis buffer) was added to the samples and incubated on a rotator overnight at 4°C. After a first centrifugation step (2,000 *g*, 4°C, 1 min), beads were washed four times with 500 μl of NP-40 lysis buffer. Protein complexes were eluted by boiling beads in 110 μl of 2 × SDS Laemmli sample buffer and 10% were analyzed by Western blotting after SDS-PAGE, as well as lysate controls.

### Mass Spectrometry Experiments (Sample Preparation and Fractionation)

DUB-probe immunoprecipitated sample eluates were diluted to 175 μl with ultra-pure water and reduced with 5 μl of DTT (200 mM in 0.1 M Tris, pH 7.8) for 30 min at 37°C. Samples were alkylated with 20 μl of iodoacetamide (100 mM in 0.1 M Tris, pH 7.8) for 15 min at room temperature (protected from light), followed by protein precipitation using a double methanol/chloroform extraction method (Wessel and Flugge, 1984). Protein samples were treated with 600 μl of methanol, 150 μl of chloroform, and 450 μl of water, followed by vigorous vortexing. Samples were centrifuged at 17,000 *g* for 3 min, and the resultant upper aqueous phase was removed. Proteins were pelleted following the addition of 450 μl of methanol and centrifugation at 17,000 *g* for 6 min. The supernatant was removed, and the extraction process was repeated. Following the second extraction process, precipitated proteins were re-suspended in 50 μl of 6 M urea and diluted to <1 M urea with 250 μl of 20 mM HEPES (pH 8.0) buffer. Protein digestion was carried out by adding trypsin (from a 1 mg/ml stock in 1 mM HCl) to a ratio 1:100, rocking at 12 rpm and room temperature overnight. Following digestion, samples were acidified to 1% trifluoroacetic acid and desalted on C18 solid-phase extraction cartridges (SEP-PAK plus, Waters), dried, and re-suspended in 2% acetonitrile and 0.1% formic acid for analysis by LC-MS/MS as described below.

Off-line high-pH reverse-phase prefractionation was performed in a similar fashion as in Davis et al. (2017). Briefly, digested material was fractionated using the loading pump of a Dionex Ultimate 3000 HPLC with an automated fraction collector and a Waters Acquity UPLC Peptide BEH C18, 300 Å, 1.7 μm, 1 mm × 100 mm (part no. 186005593) column over a 65 min gradient using basic pH reverse-phase buffers (A: water, pH 10 with ammonium hydroxide; B: 90% acetonitrile, pH 10 with ammonium hydroxide). The gradient consisted of a 15 min wash with 2% B, then increasing to 35% B over 30 min, with a further increase to 95% B in 0.1 min, followed by a 9.9 min wash at 95% B and then returning to 2% in 0.1 min, followed by re-equilibration at 2% B for 9.9 min, all at a flow rate of 100 μl/min with fractions collected every 1 min from 0 to 60 min. One hundred microliters of the fractions was dried and resuspended in 20 μl of 2% acetonitrile/0.1% formic acid for analysis by LC–MS/MS. Fractions were loaded on the LC–MS/MS following concatenation of 60 fractions into 10, combining fractions in a 10-fraction interval (F1 + F11 + F21 + F31 + F41 + F51… to F10 + F20 + F30 + F40 + F50 + F60).

### Liquid Chromatography–Mass Spectrometry/Mass Spectrometry (LC-MS/MS)

LC-MS/MS analysis was performed using a Dionex Ultimate 3000 nano-ultra high-pressure reverse-phase chromatography coupled on-line to a Q Exactive HF mass spectrometer (Thermo Scientific) as described previously (Fye et al., 2018). In brief, samples were separated on an EASY-Spray PepMap RSLC C18 column (500 mm × 75 μm, 2 μm particle size, Thermo Scientific) over a 60 min gradient of 2–35% acetonitrile in 5% dimethyl sulfoxide (DMSO), 0.1% formic acid at 250 nL/min. MS1 scans were acquired at a resolution of 60,000 at 200 m/z and the top 12 most abundant precursor ions were selected for high collision dissociation (HCD) fragmentation.

### Transcriptomics Analysis of MCF7 Cells

A total of 5 × 10^8^ MCF7 cells were grown to ~90% confluency as described above, harvested by centrifugation at 1,500 rpm, and resuspended in ice-cold PBS, centrifuged again, and pellets were kept at −20°C until analysis. RNA was extracted from cell pellets using the RNeasy Mini Kit (QIAGEN) according to the manufacturer's instructions. mRNA material was further enriched using poly-T oligo column (Manufacturer). The quality of the mRNA was checked by OD_260/280nm_ ratio and found to be ~2. The cDNA library was prepared using a standardized protocol followed by paired end sequencing using a HiSeq4000 platform (Illumina) at the Oxford Genomics Center (Wellcome Trust Center for Human Genetics, Oxford, UK).

### Transcriptomics and Proteomics Data Analysis

For the analysis of transcriptomics data, FASTQ files were converted to Binary-sequence Alignment Format (BAM) files using HISAT2 (v2.1.0) and Samtools (v1.3). Subsequently, BAM files were imported into Perseus software (v1.6.0.2) and genome annotation was performed using the Human Fasta cDNA database (http://www.ensembl.org). Reads per kilo per million (RPKM) values were calculated by a normalization step dividing by the sum (*Normalization*→*Divide*), followed by dividing normalized values by gene length, multiplying by 10^9^, and taking the log_2_ values (Table S1).

**Figure 1:**
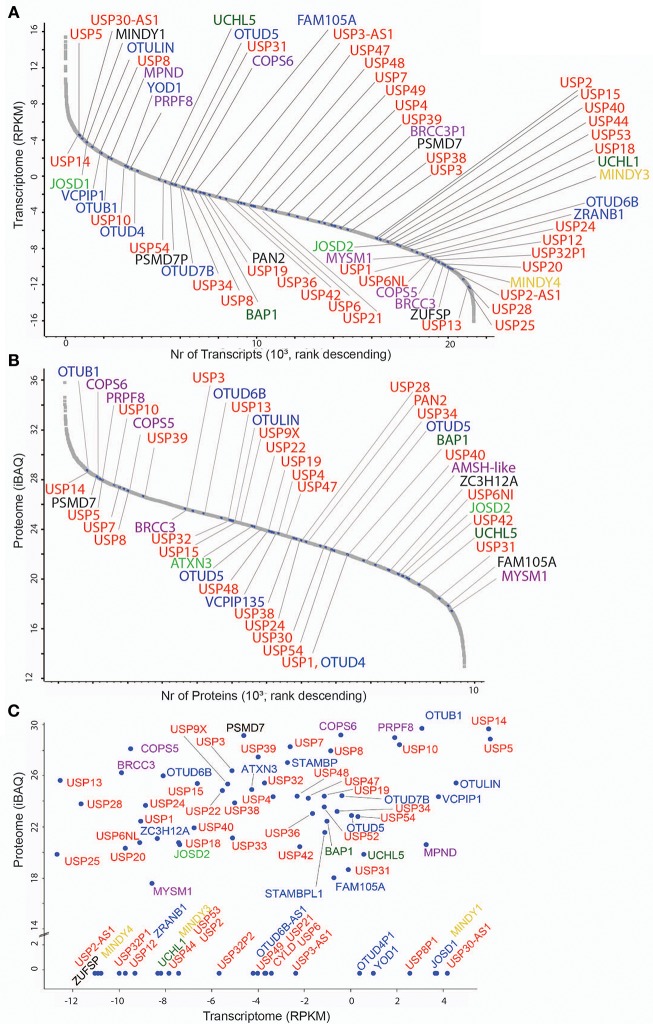
DUB transcriptome and proteome in MCF7 breast cancer cells. **(A)** Transcriptome analysis (single experiment data) listing all quantified mRNAs as reads per Kilobase per million (RPKM) values in descending values. mRNAs encoding DUBs are indicated according to their families: 56 USPs (red), 5 UCHs (dark green), 16 OTUs (blue), 11 JAMMs (purple), 4 MINDYs (yellow), 4 JOS (light green), and 1 ZUP (black). **(B)** Proteome analysis (single experiment data) listing all quantified proteins as intensity-based absolute quantitation (iBAQ) abundances in descending values. DUBs are indicated and colored based on sub-families as stated above. **(C)** Scatter Plot showing mRNA (transcriptome, *X*-axis) and protein (proteome, *Y*-axis) levels of DUBs (indicated in colors according to sub-families).

For the analysis of proteomics data, the DUB proteome in MCF7 cells was assessed by interrogating the quantified iBAQ values taken from our previous study (Davis et al., 2017) that were then matched to the MCF7 transcriptome using Perseus software (v1.6.0.2) (Table S2). For the DUB activitome, all raw MS data files from the HA-IP and high-pH fractionation experiments were analyzed in a combined fashion using MaxQuant (v1.5.5.1) and searched against the UniProt Human database (92,954 entries). Intensity values were used to compare against the DUB proteome and transcriptome (Table S3). Zero values were replaced with the value of 1 to allow for displaying the data using scatter plots as shown in Figures 1C, 7. For searches of PTMs, in particular the HA-UbPA probe adduct (112.06 Da) on cysteine residues, each raw MS data file obtained per high-pH fraction analyzed by LC-MS/MS was analyzed using PEAKS software (v 8.5; we used a 1% FDR at protein and peptide level in PEAKS with the −10LogP values of 40 for protein and 23.2 for peptide positive identification) and searched against the UniProt database (UPR_HomoSapiens_20170215). Search parameters were the following: parent mass error tolerance: 10 ppm; fragment mass error tolerance: 0.05 Da; precursor mass search: monoisotopic; enzyme: trypsin; missed cleavages: 2; variable modifications (PEAKS PTM, only the most common listed): deamidation (NQ), oxidation (M), carbamidomethylation (C), acetylation (K), acetylation (N-term), PA Probe adduct (C), maximal variable PTM per peptide: 3.

### Generation of the Human DUB Phylogenetic Tree

Genes included in this analysis:

[(CYLD, PAN2, USP17L24, USP1, USP2, USP3_H0YMI, USP3_Q9Y6I, USP4, USP5, USP6, USP7, USP8, USP9X, USP10, USP9Y, USP11, USP12, USP13, USP14, USP15, USP16, USP17L1, USP17L2, USP18, USP19, USP20, USP21, USP22, USP24, USP25, USP26, USP27X, USP28, USP29, USP30, USP31, USP32_K7EK, USP32_Q8NF, USP33, USP34, USP35, USP36, USP37, USP38, USP39, USP40, USP41, USP42, USP43, USP44, USP45, USP46, USP47, USP48, USP49, USP50, USP51, USP53, USP54), (BRCC3, COPS5, COPS6, EIF3F, EIF3H, MPND, MYSM1, PRPF8, PSMD7, PSMD14, STAMBP, STAMBPL1), (ALG13, OTUB1, OTUB2, OTUD1, OTUD3, OTUD4, OTUD5, OTUD6A, OTUD6B, OTUD7A, OTUD7B, OTULIN, OTULINL, TNFAIP3, VCPIP1, YOD1, ZRANB1), (UCHL1, UCHL3, UCHL5, BAP1), (ATXN3, ATXN3L, JOSD1, JOSD2), (MINDY1, MINDY2, MINDY3, MINDY4)].

The full-length protein sequences for each DUB were extracted from UniProt (https://uniprot.org/). The canonical sequence for each DUB was used as it was determined by UniProt. The protein alignment was performed using MUSCLE (multiple sequence alignment with high accuracy and high throughput). Finally, a constraint ML phylogenetic tree was generated by RAxML (https://raxml-ng.vital-it.ch/#/). The constraint tree was created by including DUBs into the seven known families. The LG Substitution matrix was used. The best fit model tree was further designed initially in the iTOL INTERACTIVE TREE OF LIFE (https://itol.embl.de/) where branched length was ignored, and an unrooted tree style was formed. Finally, the Adobe Illustrator software was then used to finalize the tree.

### Data Availability

MCF7 RNA-seq data have been submitted to GEO with the accession number GSE134954.

The mass spectrometry proteomics data have been deposited to the ProteomeXchange Consortium *via* the PRIDE (Perez-Riverol et al., 2019) partner repository with the data set identifier PXD014391.

## Results and Discussion

### DUB mRNA and Protein Expression Topology in MCF7 Breast Cancer Cells

To set a baseline, we wished to interrogate the number of DUBs and their abundance at the mRNA and protein level in MCF7 cells, a cell line originally established from the pleural effusion of a 69 year-old woman with metastatic disease (Brooks et al., 1973) and used for breast cancer research for more than 40 years (Comsa et al., 2015). Based on previous studies from our lab and others (Borodovsky et al., 2002; Altun et al., 2011; Turnbull et al., 2017), it appears that MCF-7 has a similar DUB profile to other immortalized cell lines but there are specific DUBs that are expressed in some cell lines and not in MCF-7. For instance, neuronal cells have high levels of active UCHL-1 [also seen to vary considerably in immortalized B-cell lines (Ovaa et al., 2004)], and HEK293 cells (human embryonic kidney) express a recently discovered DUB called ZUP1 (ZUFSP), whereas we were not able to detect either, UCHL-1 or ZUP1, in MCF-7 cells. Therefore, we feel that using the immortalized breast cancer cell line MCF7, we represent most of the endogenous DUBome. To obtain maximal depth, we performed RNA-Seq and pre-fractionation-based deep proteomics, resulting in 21,352 transcripts and 13,728 identified protein groups from which 8,949 were assigned to genes (Davis et al., 2017). In these data sets, we detected 78 DUBs by RNA-seq (76% out of 102 assigned DUBs in the human genome) (Figure 1A) and 65 DUBs at the protein level (corresponding to 53 genes) (Figure 1B). Interestingly, the DUBs MINDY1/3/4, ZUFSP, UCHL1, and alternative isoforms of OTU and USP subsets were detected only at the mRNA level, whereas others such as USP35, USP30, USP16, UCHL-3, and up to 12 DUBs were present as proteins only (Figure S1), suggesting distinct regulatory mechanisms and/or stability of their mRNA vs. protein. Generally, global mRNA expression levels poorly correlated with protein levels (Figure 1C), a trait observed in previously reported studies (Maier et al., 2009; Schwanhausser et al., 2011; Wang et al., 2019).

### Reactivity of Different Ub Probes Affects Dubome Selectivity

**Figure 2:**
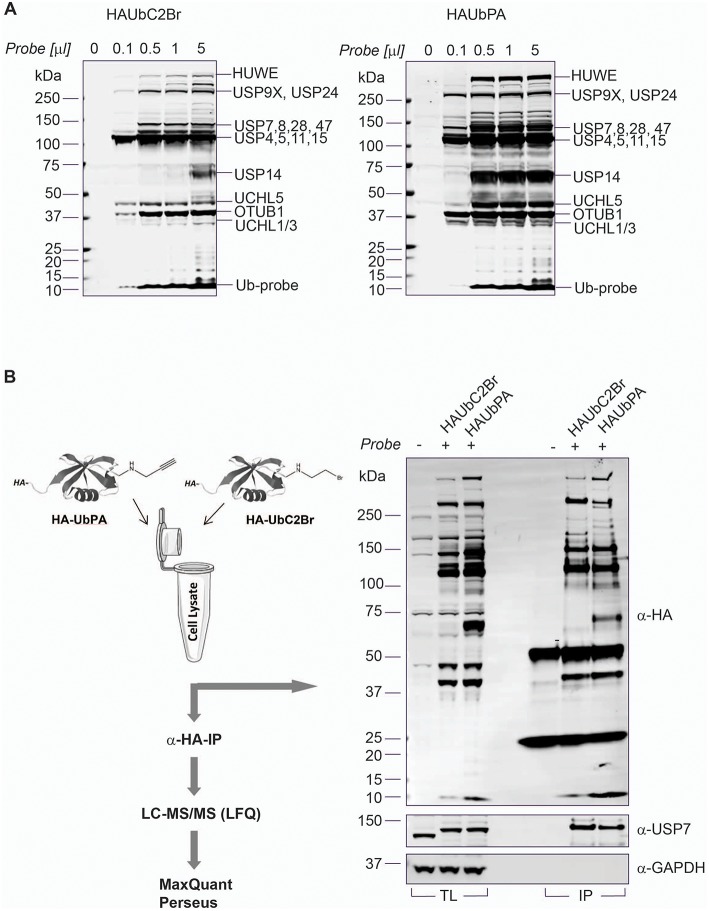
Ub-PA probe chemistry extends DUBome activity-based profiling. **(A)** Titration of HA-UbC2Br (left panel) and HA-UbPA probe (right panel) in MCF7 breast cancer cell extracts, followed by SDS-PAGE separation and analysis by anti-HA immunoblotting. Bands correspond to either DUB-probe or E3 ligase-probe adducts as indicated [based on (Altun et al., 2011) and this study]. **(B)** Left panel: Chemoproteomics workflow for profiling the active DUBome. HA-UbC2Br or HA-UbPA probe is incubated with MCF7 breast cancer cell extracts, followed by anti-HA immunoprecipitation, elution, in-solution trypsin digestion, and label-free quantitative analysis (LFQ) by LC-MS/MS. Right panel: Comparison of HA-UbC2Br and HA-UbPA immunoprecipitated DUBs analyzed by SDS-PAGE and anti-HA, anti-USP7 (positive control), and anti-GAPDH (loading control) immunoblotting.

To better gauge DUB cellular function, we aimed to match DUB expression with their activity at a global level using an improved activity-based protein profiling (ABPP)-based workflow (Altun et al., 2011; Turnbull et al., 2017). A limitation for these ABPP studies has been the proteomic technique by itself as well as some DUB targeting selectivity based on the chemical moiety of the probe (Borodovsky et al., 2002). Recently, more sensitive mass spectrometry and the use of reactive-site-centric Ub probes with a vinyl sulfone (VS) or a vinyl methyl ester (VME) revealed less DUB selectivity dependent on the chemical group used, but has also shown that some of them tend to react with non-catalytic cysteine residues, whereas propargylamide (PA)-based probes seem to react more specifically with catalytic cysteines (Hewings et al., 2018). This could be due to the unconventional reactivity of the alkyne in the propargylamide with the thiol group on the catalytic cysteine, which is unusual as electron-rich alkynes are generally poor electrophiles. UbPA reacts with DUB cysteines *via* direct addition to the terminal alkyne to give a vinyl thioether through possible radical-based intermediates (Ekkebus et al., 2013). HA-UbPA is reported to be highly DUB selective, with the exception of the additional labeling of the E3 Ub ligase HUWE1 (Ekkebus et al., 2013). We decided to compare side by side Ub probes with the different reactivities by performing an ABP assay in MCF7 cell lysates, UbPA (direct addition), UbVME (conjugate addition), and UbC2Br (nucleophilic displacement) (Figure S2A). As previously described, the UbC2Br probe has a different reactivity profile to cellular DUBs when compared to the VME probe (Borodovsky et al., 2002), in particular with the band corresponding to OTUB1 (theoretical molecular weight: 31,284 Da, ~37 kDa marker). On the other hand, USP14 (theoretical molecular weight: 56,069 Da, below the 75 kDa marker) is labeled more efficiently by UbVME as compared to UbC2Br. We concluded that the UbPA probe seems to react more efficiently with all cellular DUBs as judged by the visualized bands. To further compare the two probes with the broadest labeling profiles, we performed an ABPP by immunoprecipitating HA-tagged UbPA and UbC2Br probes after labeling MCF7 crude extracts, confirming the greater breadth of cellular DUB labeling with the UbPA probe (Figure 2A). We also analyzed probe-captured material by quantitative mass spectrometry (Figure 2B and Table S4). Both Western blotting and mass spectrometry results confirm the superior reactivity of the UbPA probe over the UbC2Br (Figure 2A and Figure S2A), being able to identify 44 DUBs with the former and 37 with the latter (Figure S2B and Table S4).

### Improved ABP Using Advanced Proteomics Methodology Expands the Dubome

**Figure 3:**
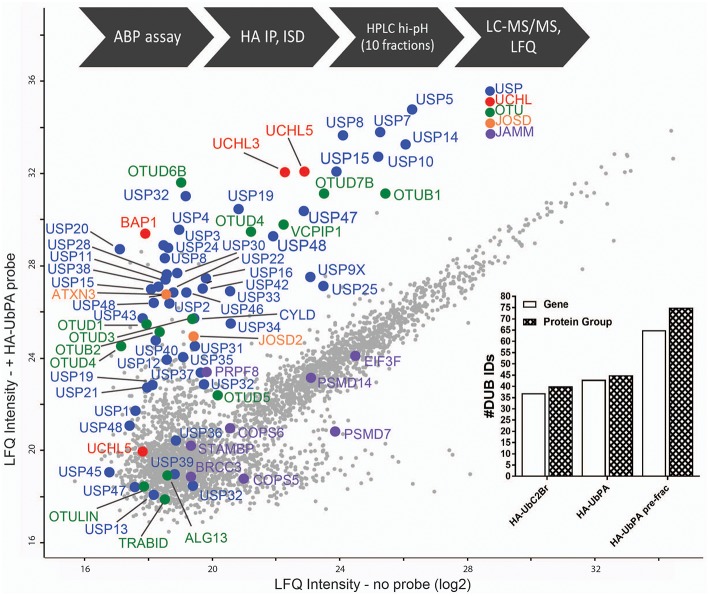
Full DUBome coverage by advanced chemoproteomics. Scatter Plot showing enrichment of DUBs upon labeling and isolation of HA-UbPA activity-based probe pulldown and quantitative analysis by mass spectrometry (concatenation of 60 fractions into 10). *X*-axis—no probe; *Y*-axis—with probe. The experimental workflow is shown on the top. The graph inclusion shows the number of DUBs captured by the HA-UbPA probe with (single experiment data) and without (technical triplicates) high-pH prefractionation post-HA-IP and digestion.

Since the number of identified DUBs using UbPA in ABPP, although improved, was still not in the range of DUBs expressed in MCF7 cells (Figure 1), we decided to explore a more advanced proteomic methodology in order to expand the active DUBome. To this end, we implemented a high-pH pre-fractionation in our classical ABPP workflow after the digestion step (Figure 2B) in order to reduce the sample complexity in an orthogonal dimension prior to LC-MS/MS analysis (Wang et al., 2011; Davis et al., 2017). This yielded an increase in the number of DUB identifications from 39 to 74 protein groups, corresponding to 65 DUB genes, greatly expanding the number observed in the conventional ABPP-MS workflow (>92%) [Figure 3 (inset) and Figure S2B]. The number of DUBs detectable *via* the ABP-MS assay is now comparable to the number of expressed DUBs in the same cell line.

### Discrimination Between Active Dubs vs. Non-active Dubs

**Figure 4:**
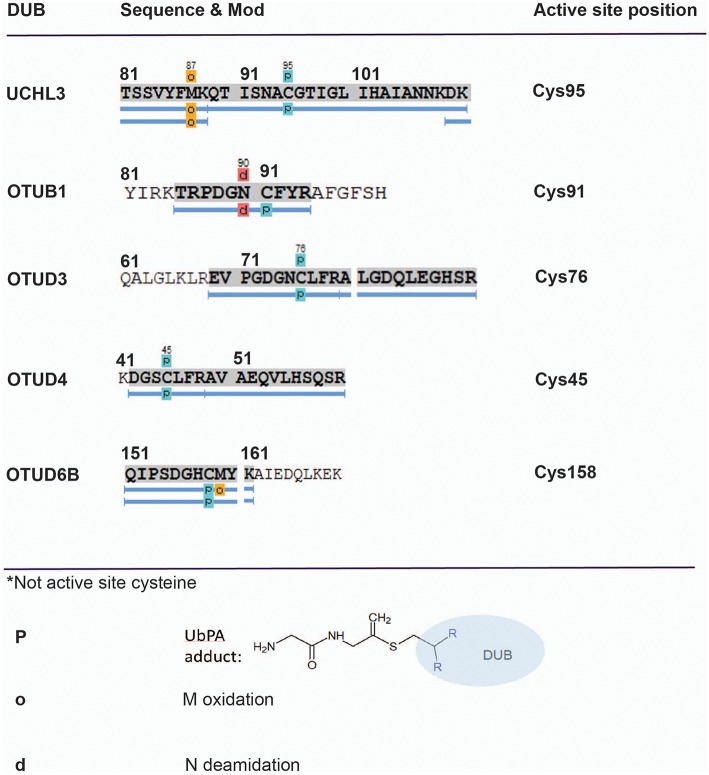
Mapping direct Ub–probe DUB adducts by mass spectrometry. LC-MS/MS analysis of HA-UbPA-labeled DUBs isolated from MCF7 breast cancer cell extracts. Peptide mapping using PEAKS analysis reveals direct cysteine–probe adducts (light blue boxes—P) for the DUBs UCHL3, OTUB1, OTUD3, OTUD4, and OTUD6B. The corresponding MS/MS fragmentation spectra for assigning the Cys–UbPA–probe adducts are listed in Figures S3A–S3E.

**Figure 5:**
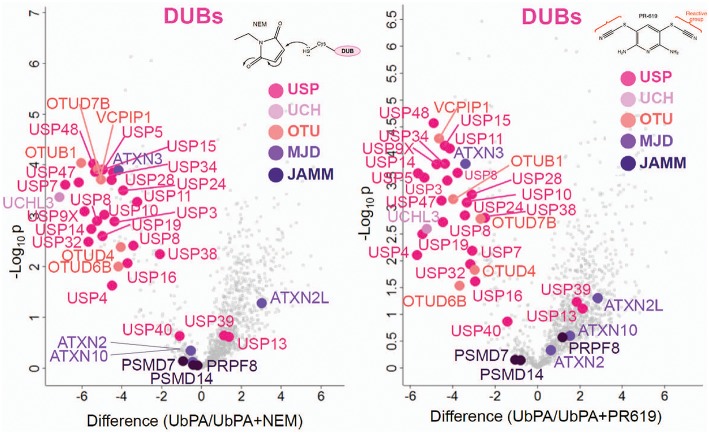
Active vs. non-active DUBs. Volcano plots showing a discrimination between cysteine-reactive and non-reactive DUBs by active-site labeling with HA-UbPA in the presence and absence of *N*-ethylmaleimide (NEM, **Left** panel) and PR-619 (**Right** panel) (data from two biological replicates run in technical duplicates). Displaced reactive DUBs are located in the upper left compartment ([probe alone]—[probe + NEM or PR619]), whereas non-reacting DUBs are left in the lower center area.

**Figure 6:**
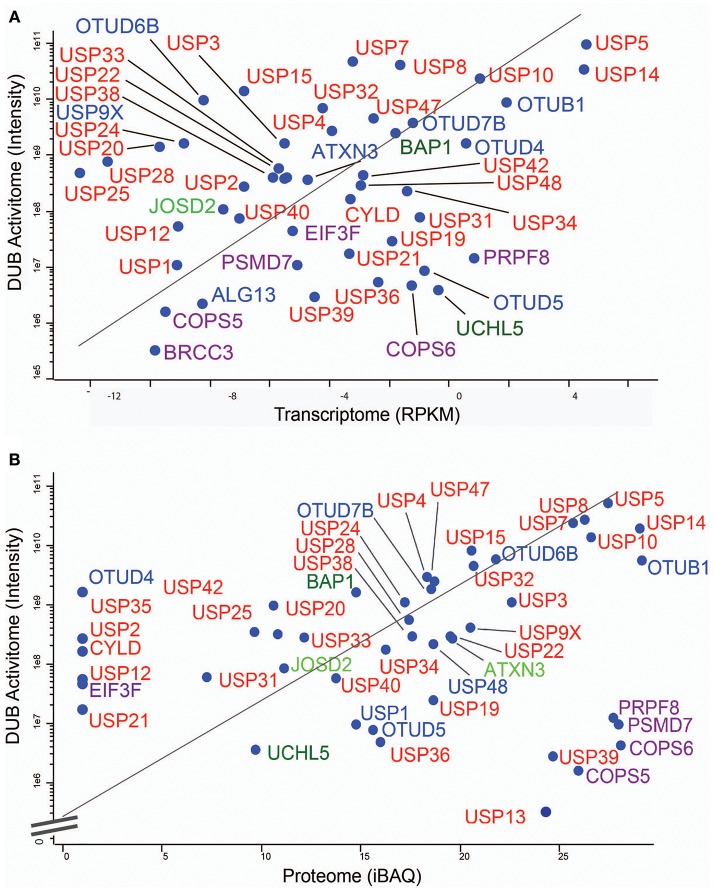
MCF7 DUB activitome vs. transcriptome and proteome. Scatter Plots showing the correlative traits of the transcriptome **(A)** and the proteome **(B)** with the DUB activitome (*X*-axis in both panels). DUBs are indicated in colors respective to their enzyme sub-families.

**Figure 7:**
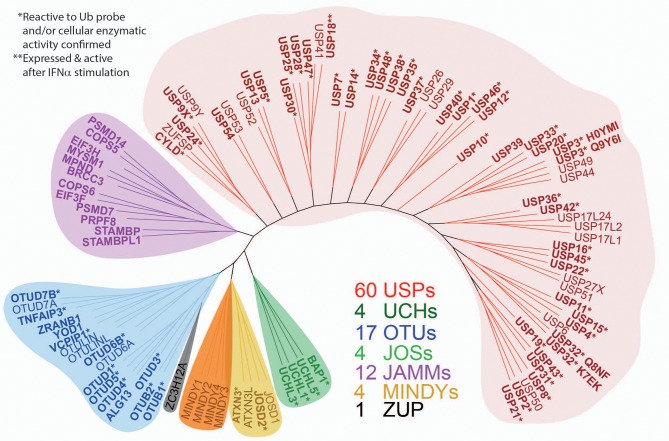
Expanded panel of active DUBs across the different enzyme subfamilies. ML (maximum likelihood) phylogenetic tree of the human DUB family. One hundred and two DUBs of the ubiquitin specific protease (USP), ubiquitin C-terminal hydrolases (UCH), ovarian tumor domain (OTU), JAMM, MINDY, JOS, and ZUP1 families are shown. In bold are the DUBs detected in the cellular proteome by active-site labeling, indicating cellular activity. *indicates inactive DUBs, in part because of mutated catalytic site cysteines and **indicates selective induction by type I interferon (IFN). The gene name nomenclature was used for consistency, but many DUBs have alternative names: ZA20D1 (OTU7B), YOD1 (OTU1/DUBA8), TNFAIP3 (OTUD7C/A20), AMSH (STAMBP1), AMSH-like (STAMBPL1), TL132 (USP32P2), TL132-like (USP32P1), LOC339799 (EIF3FP3), ZRANB1 (TRABID), PSMD14 (RPN11/POH1), USP17L2 (DUB3), OTUD6B (DUBA5, CGI-77), and PAN2 (USP52). In bold are those DUBs detected in the proteome, and the (*) indicates DUBs reactive to UbPA probe.

To gain more detailed information about cellular DUB activity captured by our ABPP assay, it was necessary to determine which fraction of enzymes directly reacted with probe and were not enriched only through affinity binding. We addressed this through two experimental approaches. First, we interrogated our data for the presence of Ub-probe adducts, which confirmed the direct reactivity of UCHL3 Cys95, OTUB1 Cys91, OTUD3 Cys76, OTUD4 Cys45, and OTUD6B Cys158 (Figure 4 and Figure S3). MS/MS analysis revealed potentially more UbPA probe adduct sites, also on non-catalytic Cys residues as well as non-DUB proteins, but systematic manual inspection of these revealed insufficient confidence of assignment. It appears that the reactivity of the probe propargyl moiety within DUBs critically depends on the correct positioning identical to the scissile isopeptide bond, which reduces potential “off-target” reactions observed with Ub-probes carrying Michael acceptors or alkyl halides (Hewings et al., 2018). Despite the clear assignment for some DUBs, this approach did not yield a comprehensive overview of DUBs reactive to probe as there were experimental limitations in the detection of tryptic peptides harboring the DUB's catalytic Cys residue, as for most DUBs, the peptide length is unsuitable for LC-MS/MS detection. To overcome this, probe variants with redox release mechanisms have been developed to selectively release probe reactive DUBs, but this was also restricted to a subset of DUBs (de Jong et al., 2017). In our case, we reasoned that probe reactive DUBs could be displaced through a direct competition using cysteine-reactive agents such as N-methylmaleimide (NEM) or the pan-DUB inhibitor PR-619 (Altun et al., 2011; Kramer et al., 2012). DUB-probe competition could be captured by a quantitative ABP-MS experiment. To this end, ABP assays were performed using HA-UbPA probe exposed to MCF7 cell extracts previously treated with excess NEM, PR-619, or DMSO control, followed by enrichment of labeled DUBS and quantitative LC-MS/MS analysis (Figure 5). Most cysteine protease DUBs were competed by NEM and PR-619, indicated by their location on the left in the volcano plot with the exception of USP13, USP39, and USP40. As expected, DUB members of the JAMM family were not affected and therefore not displaced. Interestingly, we observe that components of the 26S (e.g., PSMD7) and ATXN network (ATXN2/2L/10) were unchanged, although DUBs that are part of these complexes such as USP14 and ATXN3 are competed away, suggesting flexible complex dynamics. As a specificity control, we did not observe competition of other non-Ub cysteine proteases within the same experimental conditions (Figure S4). We concluded that some USPs may have low or no enzymatic activity under these cellular conditions, as can be monitored by our ABPP assay. For instance, USP13 may not directly react to the UbPA probe *via* an active cysteine and that it only binds *via* the Ub scaffold in a non-covalent fashion. Our results suggest that, at least under these circumstances, USP13 appears to be mostly inactive (at least toward the HA-UbPA probe) in an endogenous context where cells are not activated in a particular way, although USP13 was shown to deubiquitylate RAP80 in the context of the DNA damage response (Li et al., 2017). USP39 is a DUB in which the catalytic residue Cys 234 is replaced by an Asp, His 513 by Ser, and Asp 530 by a Glu. Its role in pre-mRNA splicing and regulation of Aurora mRNA seems therefore not dependent on catalytic activity (van Leuken et al., 2008). USP40 appears to be catalytically inactive *in vitro* despite having all the catalytic residues proposed to be important for the catalytic activity (Quesada et al., 2004). To gain further insight, we examined how activity correlates with expression. To do this, we compared the different data sets (Figure 6). DUBs identified from 5,761 quantified protein groups that directly matched to genes and mRNA transcripts (Table S1) were compared to the expanded DUB “activitome” (Table S2), resulting in a three-way comparison of DUB probe-based activity levels with their mRNA (Figure 6A) and protein level (Figure 6B and Table S3). Interestingly, DUB proteome intensities correlated relatively well with the ones from the active DUBome. This is perhaps due to the high affinity of the probe to the target, meaning that more abundant enzymes will have better/faster access to the probe, and they will probably react with it even when their activity is not as high as other less abundant enzymes. Comparing our expanded ABPP approach to the DUB proteogenomic data sets, we could identify six DUBs that were not present in the deep proteome data (CYLD, OTUD1, OTUD7A, USP12, USP2, and USP45; Figures S1, S2B), highlighting the importance of studying the active DUBome over regular expression studies. On the other hand, no members of the MINDY and ZUFSP families and most metalloprotease DUBs (JAMMS) are present in our active DUBome data. JAMMs DUBs are not supposed to react with the ABP probes utilized due to the incompatibility of the reaction mechanism of the probe to metalloproteases, and MINDY and ZUFSP proteins do not seem to be expressed in the studied cell line. Since adding a pre-fractionation step helped to get a better representation of the DUBs, we also were able to obtain information about different DUB isoforms. For instance, for USP28, three isoforms produced by alternative splicing have been described (UniProt), from which unique tryptic peptides were assigned to isoform 1 and 2 (Figure S5). For OTUD4, four isoforms have been described to be produced by alternative splicing (UniProt). We have detected unique peptides for isoforms 1 and 2, suggesting that they are expressed at the protein level. Interestingly, at the transcriptomics level, an OTUD4P1 pseudogene was detected at high levels, but not the original OTUD4 mRNA (Figure 1 and Table S1). USP15 has been characterized as expressed in four isoforms, from which isoforms 2 and 4 were confirmed at the protein level. USP47 isoform 1 shares peptides with the other forms and so was not distinguishable, but transcriptomics confirms expression of its cognate mRNA (Table S1). USP48 is expressed as eight isoforms generated by alternative splicing, from which we confirm the detection of unique peptides corresponding to isoform 1 and a shorter version referred to as A0A0A0MRS6-1 (UniProt) (Figure S5). The biological role of these isoforms is not currently understood, but mutations in USP48, potentially affecting the different isoforms differently, have been associated with Cushing's disease (Chen et al., 2018). The function of different DUB isoforms can be quite distinct, such as USP35, whose isoform 1 is an anti-apoptotic factor that inhibits staurosporine- and TNF-related apoptosis-inducing ligand (TRAIL). In contrast, USP35 isoform 2 associates to the endoplasmic reticulum (ER) and is also present at lipid droplets (Leznicki et al., 2018). Another case is USP7, for which two isoforms USP7/USP7S were described that differentially bind to Herpes virus protein and are also phosphorylated not in the same manner, affecting the degradation rate of USP7S independently of USP7 (Khoronenkova et al., 2012). For neither USP35 nor USP7 can we detect unique peptides that would discriminate between these isoforms at the protein level. Together, when we combine the sets of DUB proteomic, transcriptomic, and activitomic profiles, we are extending the global cellular DUB landscape in terms of expression and evidence for enzymatic activity (Figure 7). Generally, DUB active-site labeling was better reflected by protein abundance as compared to mRNA levels. Marked exceptions were OTUD4, USP35, USP2, USP21, and CYLD that were all detected by probe labeling, but not at the protein level, indicating very low levels of expression. The extended ABPP profiles provide deeper insights, such as detecting low abundant DUBs, such as USP2, USP21, USP12, USP46, and USP35 that have previously been challenging to be within the detection range (Table S3 and Figure S2B). Our study sets the framework for a better understanding of how physiological and pharmacological interferences affect the DUB enzyme family and their biological pathways at a global scale. Furthermore, it will help to accelerate the development of high-throughput ABP assays with a greater breadth of selectivity panel and to monitor critical DUBs relevant for human disease, as biomarkers or targets for disease modulation in a clinical context.

## Data Availability

The datasets generated for this study can be found in the MCF7 RNA-seq data has been submitted to GEO accession number: GSE134954. The mass spectrometry proteomics data have been deposited in ProteomeXchange Consortium via the PRIDE: PXD014391.

## Author Contributions

This study was conceptualised by BK and AP-F, RF, PC, and SD generated the MCF-7 deep proteome data. RF, SD, PZ, and GB generated the MCF-7 transcriptome data. AS and AP-F performed the ABPP assays and immunoblots. HS performed the high pH fractionation during MS sample preparation for proteomics. AD generated the DUB phylogenetic tree. BK, AP-F, AS, SD, and PC performed the data analysis. ES, SM, and AP-F carried the synthesis of HA-UbC2Br and HA-UbPA ubiquitin-based ABPs (activity-based probes). AP-F and BK wrote the manuscript, and all authors commented on the text.

### Conflict of Interest Statement

The authors declare that the research was conducted in the absence of any commercial or financial relationships that could be construed as a potential conflict of interest.
