# Supplementary material for: Comprehensive Landscape of Active Deubiquitinating Enzymes Profiled by Advanced Chemoproteomics
Source: Front Chem. 2019 Aug 29;7:592. doi: 10.3389/fchem.2019.00592 (PMC6727631; doi:10.3389/fchem.2019.00592)
Supplement: Supplementary file 5 [file Data_Sheet_1.docx]

**SUPPLEMENTARY INFORMATION**

**Figure Legends**

**Figure S1: *Overlap of MCF7 transcriptome versus deep proteome*.** Venn diagram showing mRNA transcripts in blue and proteins in yellow.

**Figure S2: *Improved activity-based profiling increases cellular coverage of DUBs.* A** Comparison of the different active site probes HAUbC2Br, HAUbPA and HAUbVME. **B** Venn diagram showing overlap of cellular DUBs captured in the ABP assay using HAUbC2Br (blue), HAUbPA (yellow) and HAUbPA combined with high pH pre-fractionation (green).

**Figure S3: *MS/MS spectra showing UbPA probe – DUB adducts*. A** MS/MS spectra for the UCHL3 derived peptide _89_QTISNACGTIGLIHAIANNK_108_ detected as precursor ions M+3H^3+^ (top panel) and M+4H^4+^ (middle panel) as well as the peptide _89_QTISNACGTIGLIHAIANNKDK_110_ detected as M+3H^3+^ (lower panel), all carrying the PA adduct (+112.06Da) at Cys95. **B** MS/MS spectrum for the OTUB1 derived peptide _85_TRPDGNCFYR_94_ detected as precursor M+2H^2+^ carrying the PA adduct (+112.06Da) at Cys91. **C** MS/MS spectrum for the OTUD3 derived peptide _70_EVPGDNCLFR_79_ detected as precursor M+2H^2+^ carrying the PA adduct (+112.06Da) at Cys76. **D** MS/MS spectrum for the OTUD4 derived peptide _42_DGSCLFR_38_ detected as precursor M+2H^2+^carrying the PA adduct (+112.06Da) at Cys45. **E** MS/MS spectrum for the OTUD6 derived peptide QIPSDGHCMYK detected as precursor M+2H^2+^carrying the PA adduct (+112.06Da) at Cys158. In support of **Fig.4**.

**Figure S4: *ABP assay using HAUbPA probe does not target cysteine proteases outside the DUB family*.** Volcano plot showing differential abundance between cellular pulldowns with HAUbPA versus HAUbPA in the presence of NEM (left panel) or the pan-DUB inhibitor PR619 (right panel).

**Figure S5: *Detection of DUB isoforms at the proteome level.*** Examples are shown for which unique peptides per DUB isoform were detected, such as USP28 (P40818, P40818-2), OTUD4 (Q01804, Q01804-5), USP15 (Q9Y4E8, Q9Y4E8-2), USP47 (Q96K76, Q96K76-2, Q96K76-4) and USP48 (Q86UV5, A0A0A0MRS6). Horizontal bar graphs indicate peptide coverage (blue bars) across the protein sequence. Since these DUB isoform species were identified in the high-pH based ABP MS assay and that they all preserve the catalytic triad sequence motif, these reflect enzymatically active species.

**Supplementary Tables:**

**Table S1:** ***Quantified MCF7 full and DUB transcriptome & proteome***. Worksheet 1: All genes; worksheet 2: DUBs; worksheet 3: DUBs simple.

**Table S2:** ***HAUbPA and high pH enriched Protein groups and DUBs detected by quantitative mass spectrometry***. Worksheet 1: All protein groups; worksheet 2: DUBs.

**Table S3:** ***MCF7 expressed DUBs - Transcriptome, Proteome, Activitome.***

**Table S4:** ***HAUbC2Br and HAUbPA DUB interactome.***
