# Supplementary figures and images for: Comprehensive Landscape of Active Deubiquitinating Enzymes Profiled by Advanced Chemoproteomics
Source: Front Chem. 2019 Aug 29;7:592. doi: 10.3389/fchem.2019.00592 (PMC6727631; doi:10.3389/fchem.2019.00592)

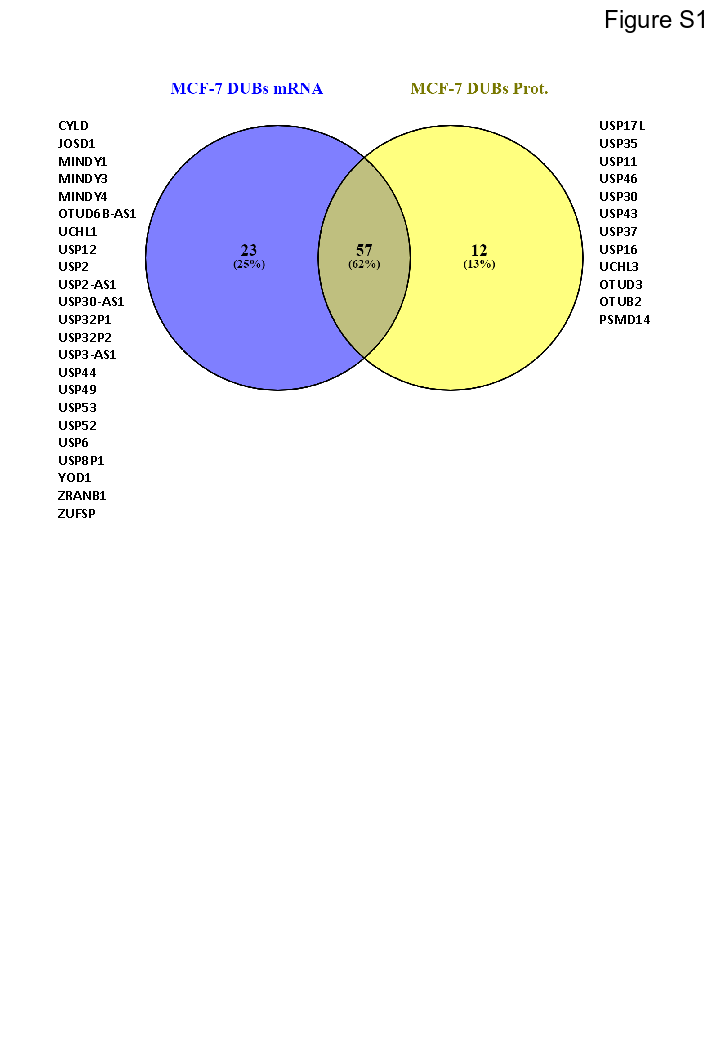

Supplement: Supplementary file 6 [file Image_1.PNG]

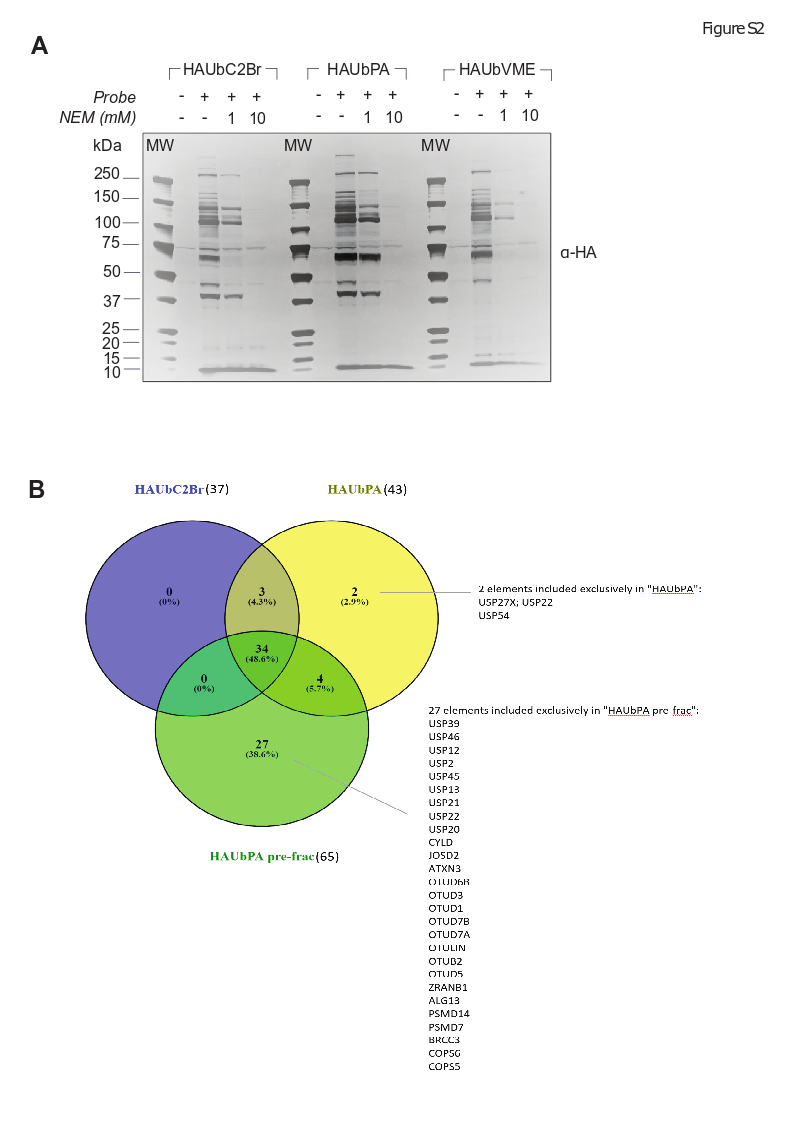

Supplement: Supplementary file 7 [file Image_2.PNG]

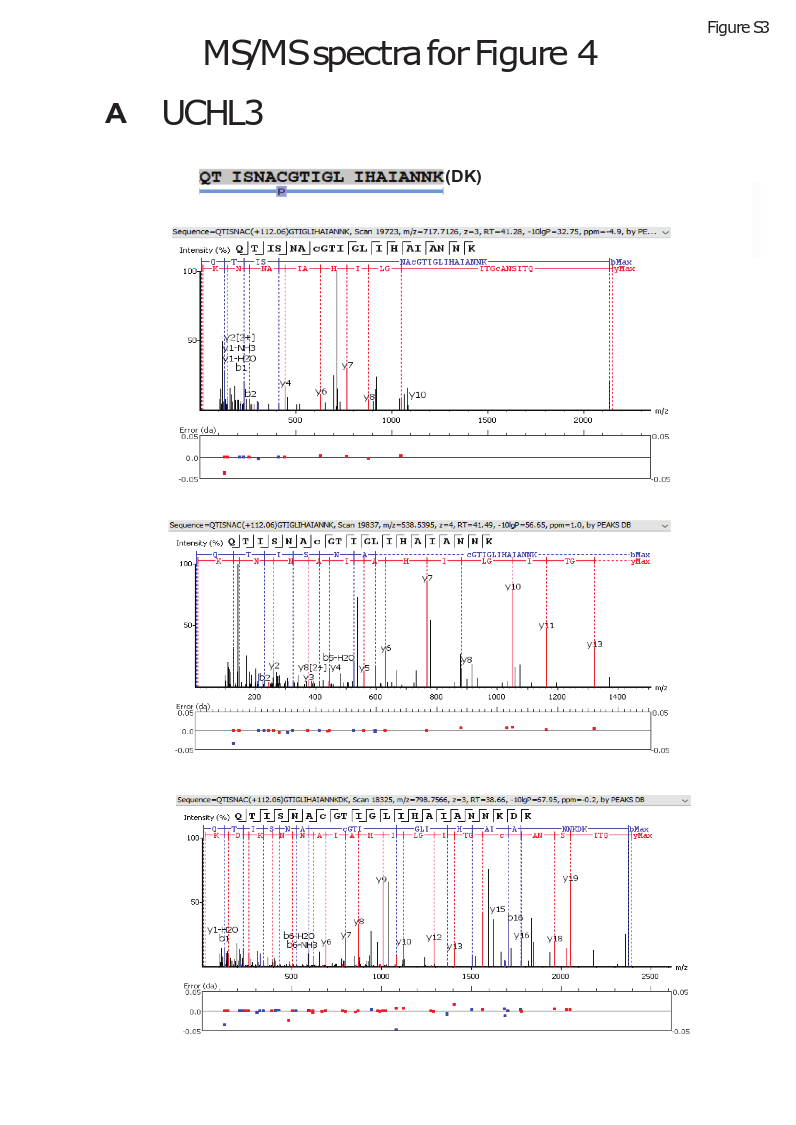

Supplement: Supplementary file 8 [file Image_3.PNG]

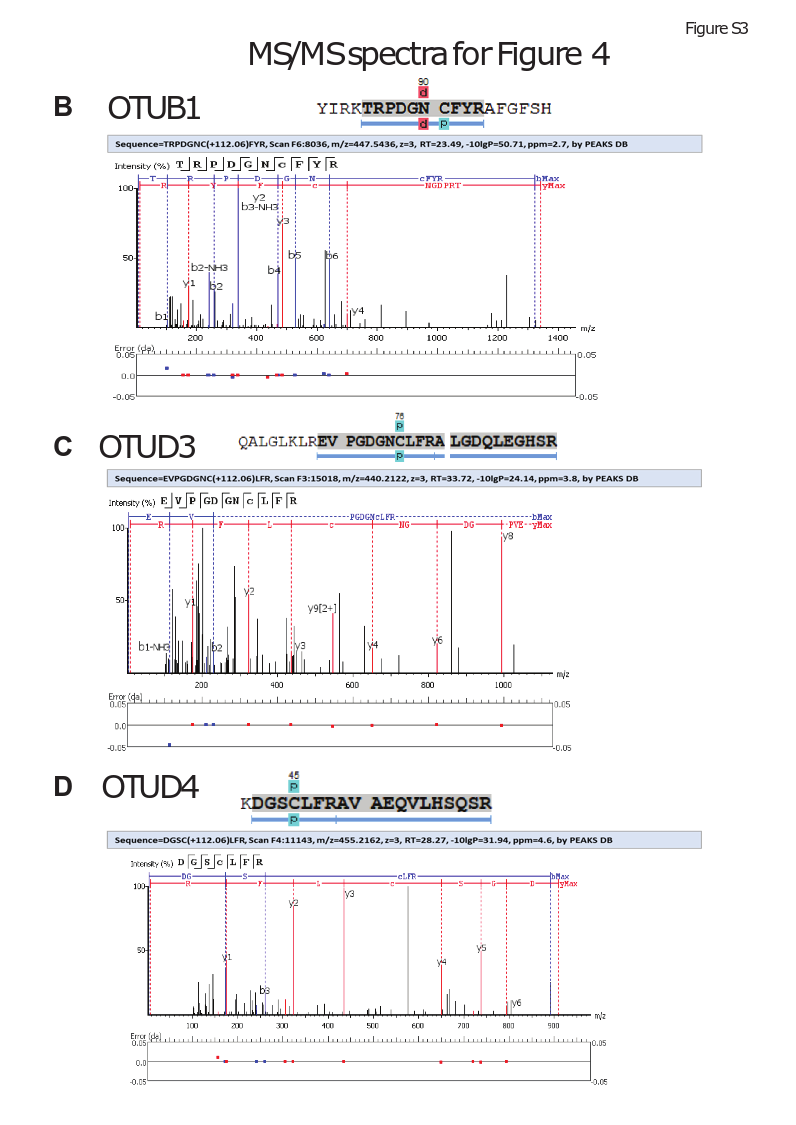

Supplement: Supplementary file 9 [file Image_4.PNG]

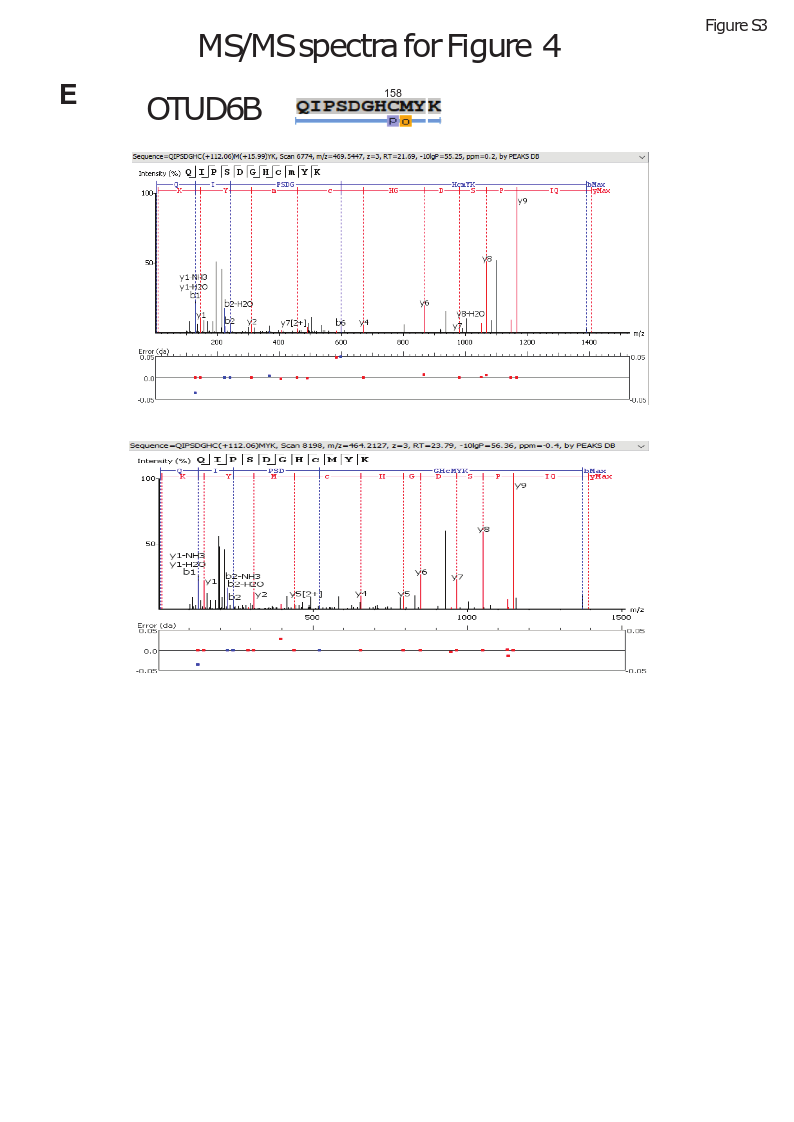

Supplement: Supplementary file 10 [file Image_5.PNG]

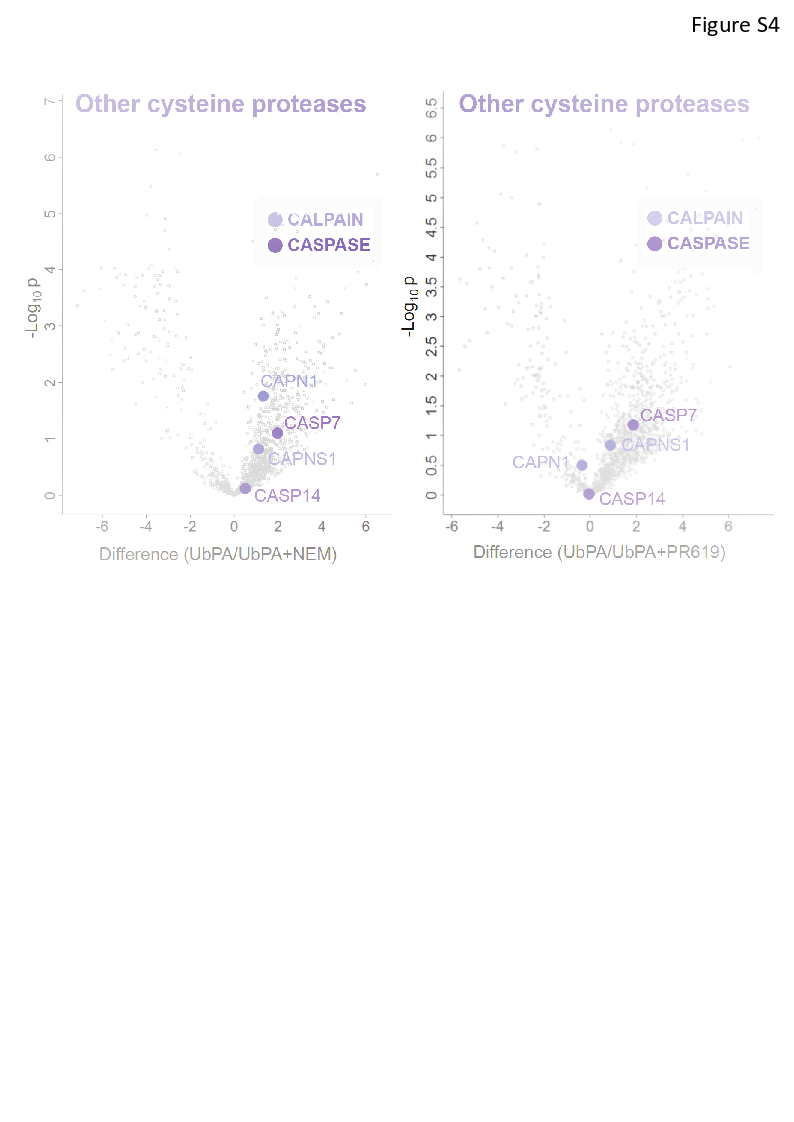

Supplement: Supplementary file 11 [file Image_6.PNG]

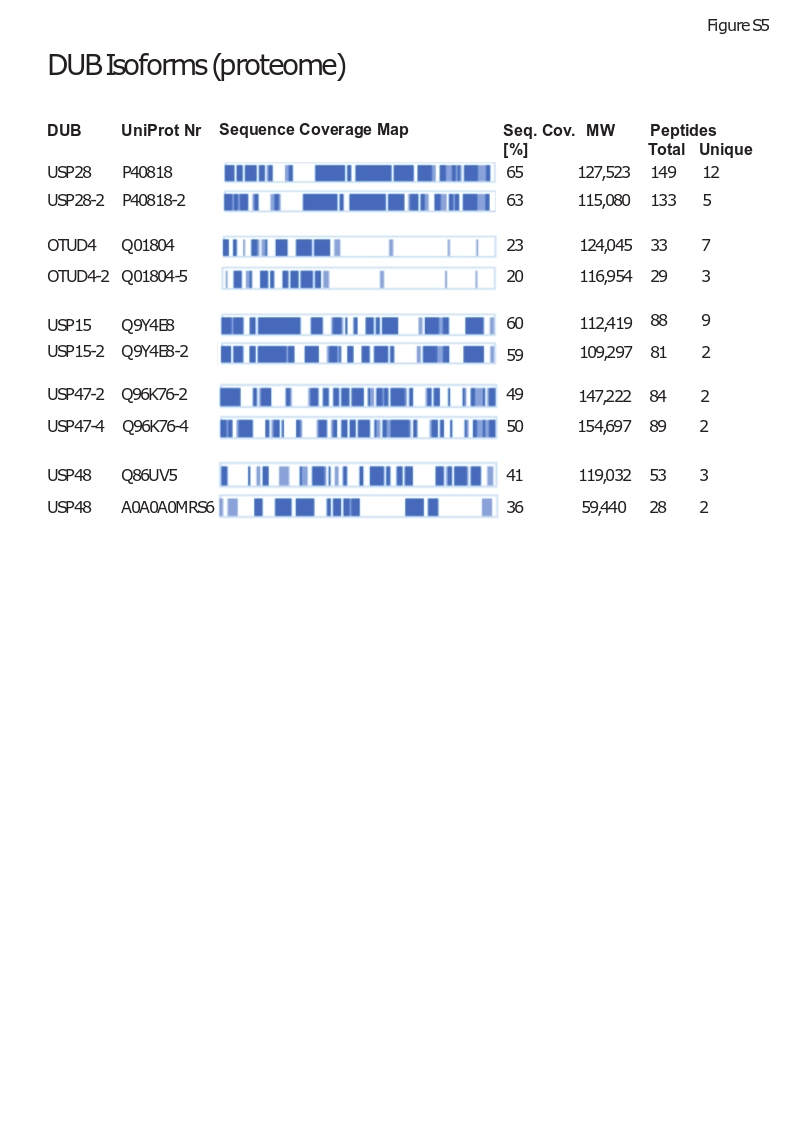

Supplement: Supplementary file 12 [file Image_7.PNG]
